# High cocoa polyphenol rich chocolate may reduce the burden of the symptoms in chronic fatigue syndrome

**DOI:** 10.1186/1475-2891-9-55

**Published:** 2010-11-22

**Authors:** Thozhukat Sathyapalan, Stephen Beckett, Alan S Rigby, Duane D Mellor, Stephen L Atkin

**Affiliations:** 1 Department of Endocrinology, Diabetes and Metabolism, Hull York Medical School, Michael White Diabetes Centre, Hull Royal Infirmary, 220-236 Anlaby Road, Hull, HU3 2RW, UK; 2 Humber Obesity, Nutrition, Education and Innovation (HONEI) Project, Room 332, Hertford Building, University of Hull, Cottingham Road, Hull HU6 7RX, UK; 3 Department of Chemistry, University of Hull, Cottingham Road, Hull HU67RX, UK; 4 Academic Cardiology and Hull York Medical School, University of Hull, Cottingham Road, Hull HU6 7RX, UK

## Abstract

**Background:**

Chocolate is rich in flavonoids that have been shown to be of benefit in disparate conditions including cardiovascular disease and cancer. The effect of polyphenol rich chocolate in subjects with chronic fatigue syndrome (CFS) has not been studied previously.

**Methods:**

We conducted a double blinded, randomised, clinical pilot crossover study comparing high cocoa liquor/polyphenol rich chocolate (HCL/PR) in comparison to simulated iso-calorific chocolate (cocoa liquor free/low polyphenols(CLF/LP)) on fatigue and residual function in subjects with chronic fatigue syndrome. Subjects with CFS having severe fatigue of at least 10 out of 11 on the Chalder Fatigue Scale were enrolled. Subjects had either 8 weeks of intervention in the form of HCL/PR or CLF/LP, with a 2 week wash out period followed by 8 weeks of intervention with the other chocolate.

**Results:**

Ten subjects were enrolled in the study. The Chalder Fatigue Scale score improved significantly after 8 weeks of the HCL/PR chocolate arm [median (range) Exact Sig. (2-tailed)] [33 (25 - 38) vs. 21.5 (6 - 35) 0.01], but that deteriorated significantly when subjects were given simulated iso-calorific chocolate (CLF/CP) [ 28.5 (17 - 20) vs. 34.5 (13-26) 0.03]. The residual function, as assessed by the London Handicap scale, also improved significantly after the HCL/PR arm [0.49 (0.33 - 0.62) vs. 0.64 (0.44 - 0.83) 0.01] and deteriorated after iso-calorific chocolate [00.44 (0.43 - 0.68) vs. 0.36 (0.33 - 0.62)0.03]. Likewise the Hospital Anxiety and Depression score also improved after the HCL/PR arm, but deteriorated after CLF/CP. Mean weight remained unchanged throughout the trial.

**Conclusion:**

This study suggests that HCL/PR chocolate may improve symptoms in subjects with chronic fatigue syndrome.

## Background

Chronic fatigue syndrome (CFS) is a debilitating condition with high morbidity and an associated reduced quality of life[1]. Surveys examining the prevalence of CFS have reported prevalence rates ranging from 0.2% to 2.6% in primary care populations[2]. Various therapies including antidepressants, cognitive behaviour therapy and graded exercise therapy have been tried in CFS with varying effects [3].

Cocoa contains a complex mixture of polyphenols especially flavonoids. Observational studies report that dietary flavonoids may reduce the mortality risk from coronary heart disease, cancer and stroke[4,5]. Experimental studies on animals and cultured human cell lines have demonstrated the role of polyphenols in the prevention of cardiovascular diseases, cancers, neurodegenerative diseases, diabetes and osteoporosis[6]. The effect of chocolate on fatigue appears to have been first described by the Aztec Emperor Montezuma II (born c.1480 reigned 1502-1520) who noted, "The divine drink, which builds up resistance and fights fatigue. A cup of this precious drink [cocoa] permits man to walk for a whole day without food''. The Badianus Codex (1552) noted the use of cocoa flowers to treat fatigue [7]. Chocolate is known to increase neurotransmitters like phenyl ethylamine, serotonin, and anandamide in the brain [8]. An imbalance in various neurotransmitters including serotonin have also been reported in subjects with CFS[9]. The effect of cocoa in subjects with chronic fatigue syndrome has not been studied to date. We hypothesised that chocolate, by modulating neurotransmitters, might reduce the symptom burden of CFS. Therefore, we conducted a double blinded, randomised, clinical pilot crossover study comparing high cocoa liquor/polyphenol rich chocolate to simulated iso-calorific chocolate (cocoa liquor free/low polyphenols) on fatigue and residual function in subjects with chronic fatigue syndrome.

## Materials and methods

Ten consecutive subjects with chronic fatigue syndrome from a secondary care setting who fulfilled the exclusion and inclusion criteria were recruited in this study. The Centres for Disease Control and Prevention criteria were used to diagnose Chronic Fatigue syndrome[10]. Subjects having severe fatigue of at least 10 out of 11 on the Chalder Fatigue Scale (binary scored) were enrolled[11].

The exclusion criteria were: any co-morbid psychiatric disorder classified according to Diagnostic and Statistical Manual of Mental Disorders (fourth edition) (DSM IV) criteria (APA 1984), subjects taking more than 10 gm of chocolate per day, use of any prescription medication including benzodiazepine or antidepressant drugs, over the counter medications including herbal remedies two months before study entry, and an inability to attend the hospital for screening tests or follow-up visits.

All participants gave their written informed consent prior to participating in the study. The study was approved by the Hull and East Riding Local Research Ethics Committee. Block randomisation was performed. Subjects had 8 weeks of the initial intervention and then 8 weeks of the crossover intervention separated by a 2 week wash out between the 2 arms. Severity of fatigue and handicap was analysed using using validated self-report questionnaires (Chalder Fatigue Scale (Likert Scored), the London Handicap Scale (LHS) and the Hospital Anxiety and Depression Scale before and after each phase of the trial [11-13].

The active product was polyphenol rich chocolate containing 85% cocoa solids (derived from a high cocoa liquor content) compared with chocolate containing cocoa butter alone that contained no non-fat cocoa solids (cocoa liquor) (simulated iso-calorific chocolate). Chocolate containing cocoa butter alone was dyed to the same colour as the high cocoa liquor/polyphenol rich chocolate (with a recipe specially prepared by Nestlé PLC York. UK). Individual 15 g foil wrapped bars were provided and subjects were asked to consume 1 bar three times daily. Subjects were reviewed by the dietician during screening, before and after each intervention to assess whether they were taking more than 10 g of chocolate per day prior to recruitment, as well as to advise them not to consume extra chocolate during the study period. Apart from this, subjects were advised not to change their diet. A taste trial was performed prior to the study to confirm that subjects could not distinguish between the two treatments.

Analysis of the chocolate was performed by Nestlé PLC York. The simulated iso-calorific chocolate with the dye contained 547 Kilocalories/100 g where as the dark chocolate with cocoa powder contained 545 kilocalories/100 g (''Genesis for Windows, Esha Research'' software). The glycaemic index of dark chocolate was 41 where as for simulated iso-calorific chocolate was 40. The composition of high cocoa liquor/polyphenol rich chocolate and the simulated iso-calorific chocolate is given in Table 1, and the polyphenol content of each shown in Table 2. The total percentage of non-fat cocoa solids (cocoa liquor) in polyphenol rich chocolate was 33.17%. Micro analyses were carried out on a cross section of samples resulting in no salmonella or enterobacter laceae.

**Table 1:** Composition of high cocoa liquor/polyphenol rich chocolate and simulated iso-calorific chocolate (cocoa liquor free/low polyphenol)

Composition by ingredient	High Cocoa Liquor Polyphenol Rich chocolate %	Simulated iso-calorific chocolate (Cocoa Liquor Free/Low Polyphenol) %
Cocoa liquor (non fat)	68.10	00.00

Sugar	22.64	43.39

Whole milk powder	00.00	27.05

Cocoa butter	06.42	20.01

Whey powder	00.00	04.95

Vegetable fat	00.00	04.34

Butter fat	02.45	00.00

Lecithin	00.34	00.21

Vanillin	00.05	00.05

**Composition by Macronutrient**	**%**	**%**

Protein	5.43	5.72

Carbohydrate	19.51	41.78

Fat	75.05	52.44

**Table 2:** Polyphenol analysis* high cocoa liquor/polyphenol rich chocolate and simulated iso-calorific chocolate (cocoa liquor free/low polyphenol)

	High Cocoa Liquor/Polyphenol Rich chocolate	Simulated iso-calorific chocolate (Cocoa Liquor Free/Low Polyphenol)
Polyphenols		

Folin	17.4 mg ECE/g	3.9 mg ECE/g

Catechin	0.37 g/kg	< 0.05 g/kg

Dimer B2	0.80 g/kg	< 0.05 g/kg

Epicatechin	1.23 g/kg	< 0.05 g/kg

Trimer C	0.58 g/kg	< 0.05 g/kg

Tetramer D	0.33 g/kg	< 0.05 g/kg

Dimer B5	0.32 g/kg	< 0.05 g/kg

*Analysed by Nestlé UK Quality Assurance Laboratory

### Statistical Methods

We calculated sample size according to previous published data on the fatigue scale in chronic fatigue syndrome[14]. With a confidence interval of 1-alpha (2 sided), and a standard deviation of 2.25, for 80% power that gave a number of 10 subjects to be studied to show a 4 point difference between the two treatments including a drop out rate of 2 subjects. Statistical analysis was performed using SPSS for Windows NT, version 9.0 (SPSS Inc., Chicago, Illinois). Data was analyzed on an 'intention to treat' basis with the Wilcoxon Signed Rank sum test before and after intervention. An arbitrary level of 5% statistical significance was assumed (two tailed). Successful demonstration of efficacy for the trial was defined as a statistically significant superiority over cocoa absent chocolate in the primary outcome variable, fatigue.

## Results

Ten subjects (6 females and 4 males) were enrolled in the study. The mean age of subjects was (mean ± SD) 52 ± 8 years and mean body mass index was 28.3 ± 2 kg/m^2^. The Chalder Fatigue Scale score improved significantly after 8 weeks of the active high cocoa polyphenol rich chocolate phase, however these scores deteriorated significantly when subjects were given simulated iso-calorific cocoa liquor free/low polyphenol chocolate (Table 3). The residual function, as assessed by the London Handicap scale, also improved significantly after the active phase and deteriorated during the placebo phase (Table 3). A similar trend was also found in HAD scale. Anecdotally, two subjects were able to return back to work after having had their symptoms for a 2 year period and chose to continue on a high cocoa solid chocolate diet.

**Table 3:** Questionnaire scores before and after active (high cocoa liquor/polyphenol rich chocolate) and placebo (simulated iso-calorific chocolate - cocoa liquor free/low polyphenol)

Questionnaires	Before Intervention	After Intervention (8 weeks)	Wilcoxon Signed Rank Sum test
	**Median (Range)**	**Median (Range)**	***Z value (Exact Sig. (2-tailed))***
Chalder Fatigue questionnaire Active Arm	33 (25 - 38)	21.5 (6 - 35)	-2.53 (0.01)

Chalder Fatigue questionnaire Placebo Arm	28.5 (17 - 20)	34.5 (13-26)	-2.23 (0.03)

HAD questionnaire (Anxiety) Active Arm	9.5 (8-13)	6 (4-8)	-2.54 (0.01)

HAD questionnaire (Anxiety) Placebo Arm	6 (3-9)	9 (8-12)	-2.21 (0.03)

HAD questionnaire (Depression) Active Arm	10 (9-14)	5.5 (4-9)	-2.68 (0.01)

HAD questionnaire (Depression) Placebo Arm	6 (4-9)	12 (8-14)	-2.34 (0.02)

LHS questionnaire (residual function) Active Arm	0.49 (0.33 - 0.62)	0.64 (0.44 - 0.83)	-2.52 (0.01)

LHS questionnaire (residual function) Placebo Arm	0.44 (0.43 - 0.68)	0.36 (0.33 - 0.62)	-2.21 (0.03)

The mean weight before and after the high cocoa chocolate intervention were unchanged at 77.04 kg and 76.80 kg, respectively (p value - 0.326). The mean weight before and after the placebo intervention were also unchanged (73.43 kg vs. 73.85 kg, respectively p value 0.345).

## Discussion

This study shows that high cocoa polyphenol rich chocolate reduced the burden of symptoms in subjects in chronic fatigue syndrome. As both types of chocolate were iso-calorific and had similar glycemic indices and loads, it is likely that the improvement was due to the high polyphenol content within the active chocolate, rather than a difference in of the micro or macronutrient the composition of the two chocolates. The significance of the results is particularly surprising because of the small number of subjects in the study.

It is also interesting to note that there was no significant weight change after both phases of the study despite consuming an extra 245 Kcal daily for each two month period. The potential weight gain may have been offset by improvement in functional status and physical activity when they had high cocoa polyphenol rich chocolate. However, this would not explain why the weight was unchanged in the placebo group, particularly as they reported an increase in their fatigue.

Arachidonyl ethanolamide, later called anandamide (a name that comes from ananda, the Sanskrit word for ''bliss'') has a structural similarity with tetrahydrocannibol, and other strongly related compounds that are found in cocoa[8]. There are also compounds (N-acylethanolamines) that block the breakdown of anandamide in cocoa[8]. It may be the synergy of these compounds in chocolate that may account for the results seen in this study.

A number of biological systems have been implicated in chronic fatigue syndrome and there is mounting evidence that oxidative stress [15-18] contributes to the disease process and to some of the symptoms in the illness [19]. Flavanoids have shown to have a protective effect on cells including neuronal cells from oxidative stress[20]; however, this mechanism needs to be explored in further studies.

Despite the positive findings, this study is still limited due the small number of participants and larger studies are necessary to confirm these findings.

As this was a proof of concept study; physical activity was not formally assessed. Biochemical markers including plasma polyphenol levels and inflammatory markers were not measured as part of the study as all of the baseline parameters prior to the study were normal, in accord with the criteria for the diagnosis of chronic fatigue syndrome.

Since there was a consistent improvement of symptoms with high cocoa phenol chocolate and deterioration with iso-calorific chocolate, a placebo effect is unlikely. Moreover, the taste panel of healthy people before the study could not differentiate the taste between high cocoa and iso-calorific chocolate.

In summary, this study suggests that consuming high cocoa polyphenol rich chocolate 15 g three times daily has a beneficial effect in improving fatigue and residual function in subjects with CFS over a period of 8 weeks compared to simulated iso-calorific cocoa mass free/low polyphenol chocolate. This may be of benefit for subjects with CFS if included as part of a healthy diet or in a formal treatment protocol.

## Competing interests

None for TS, ASR, DDM and SLA

SB worked as Head of Research for Nestlé, PLC, York

## Authors' contributions

TS, SB, ASR, DDM and SLA contributed to the design, conduct, analysis, drafting and revision of the manuscript. All authors read and approved the final manuscript.
